# Construction of a Synthetically Engineered *nirB *Promoter for Expression of Recombinant Protein in *Escherichia coli*

**DOI:** 10.5812/jjm.15942

**Published:** 2014-07-01

**Authors:** Reza Nasr, Mohammad Reza Akbari Eidgahi

**Affiliations:** 1 Semnan Biotechnology Research Center, Semnan University of Medical Sciences, Semnan, IR Iran

**Keywords:** Recombinant Proteins, Enzyme-linked Immunosorbent Assay, *Escherichia coli*, Nitrate reductase (NADH), Nitrite Reductase (NAD(P)H), Gene Expression

## Abstract

**Background:**

Anaerobic-inducible promoters are alternatives of chemical-inducible promoters for expression of recombinant proteins especially in conditions where chemical induction is not possible or anaerobic conditions are preferable. The *nirB *promoter is the promoter of the first gene of nir operon in *Escherichia coli*, which encodes NADH-dependent nitrite reductase. This promoter is naturally induced under anaerobic conditions and upregulated by nitrite and nitrate.

**Objectives:**

The current study was carried out to construct a synthetic *nirB *promoter that does not respond to chemical inducers (nitrite or nitrate), but instead responds to anaerobic induction. For this purpose, a new plasmid was constructed (pFS*nirB*78-23LTB), which contains a synthetic *nirB *promoter. The activity of this plasmid was evaluated in *E. coli* under both aerobic and anaerobic conditions and in response to chemical inducers, nitrite and nitrate.

**Materials and Methods:**

A synthetic *nirB *promoter was firstly cloned into a pKK223 derivative plasmid and then the heat labile toxin B subunit gene (LTB) of entrotoxigenic *E. coli* was cloned under the control of this promoter. The inducibility of this plasmid in *E. coli* was measured under anaerobic conditions in the presence or absence of nitrite or nitrate by ganglioside GM1 ELISA.

**Results:**

Our data showed that this promoter is strongly induced under anaerobic conditions while it showed much lower activity (11%) under aerobic conditions. In contrast to the native promoter, this promoter was not induced by chemical inducers, nitrite or nitrate.

**Conclusions:**

This study showed that the recombinant protein produced under the control of synthetic *nirB *promoter has critical characteristics such as pentamer formation, receptor recognition ability and conservation of antigenic epitopes. In addition, the data showed anaerobiosis and chemical inducers had no adverse effects on recombinant proteins. Based on the results, this synthetic promoter is suitable for use in live delivery vaccines or drug systems and for production of recombinant proteins especially oxygen sensitive proteins.

## 1. Background

Expression of recombinant proteins in *Escherichia coli* are usually performed in high oxygen tension and controlled by various chemically or physiologically inducible promoters (1, 2). Chemically inducible promoters such as *lac*, *tac*, *trc*, *trp* and *T7,* used routinely for production of recombinant proteins but they have economic and technical restrictions particularly when used for production on an industrial scale (3). They may require additional steps in the production process and the isolation of the inducer from the final product is costly. On the other hand, in case of oxygen-sensitive proteins, high O_2_ tension may have adverse effects on protein structure and function. Hence, many alternative inducible and controllable promoter systems have been investigated for recombinant protein production (3, 4). 

As transcription is basically controlled via promoter elements, promoter engineering is a fundamental factor for control of transcription and thereupon protein production (5). Anaerobically inducible promoters are an alternative of chemically inducible promoters for expression of recombinant proteins especially in conditions where chemical induction is impossible or anaerobic conditions are preferable (6). The *nirB *promoter is the promoter of the first gene of *E. coli* NADH-dependent nitrite reductase operon. This promoter is induced in anaerobic conditions and up regulated in response to nitrite and nitrate. 

There are two critical regions on this promoter for induction by anaerobic conditions. The first being a hexamer region, TAAGGT, at -10 positions that it is necessary for anaerobic activation and mutation in this region may result in loss of promoter activity. The second region is the fumarate and nitrate reductase regulatory (FNR) protein-binding site and mutation in this region results in decreasing promoter induction. FNR protein encoded by the *fnr* gene is a general regulatory protein that activates transcription initiation in some anaerobic promoters such as *nirB *under anaerobic conditions. Expression of protein under the control of this promoter is totally dependent on FNR transcription factor in the -30 and -52 region upstream to the transcription start point. The transcription start point of the *nirB *gene is positioned at the -23 or -24 region, related to the ATG initiation codon (7). 

There are also two heptamer regions about 20 bp upstream to the FNR region (-69 and -70 upstream to transcription start point), which are binding sites of two other regulatory proteins; NarP and NarL. Mutation in NarP, NarL or both decreases *nirB *promoter activity; this means that NarL and NarP are cofactors of FNR in the transcription process. It has been demonstrated mutation in the NarL binding site suppresses nitrite induction whereas it has no effect on nitrate induction (8, 9). A synthetic *nirB *promoter was successfully used for *in vitro* expression of fragment C of tetanus toxin in both *E. coli* and *Salmonella typhimurium*. This promoter also shows effective *in vivo* inducibility, in conditions where there is normally no oxygen tension (10). 

In recombinant protein expression systems, removing these inducers from culture medium result in further steps for purification and then more cost. In addition, these chemical inducers may have some side-effects on cell physiology, and since using these inducers is not applicable *in vivo*, the present study was performed to construct a synthetic *nirB *promoter, which has no responding regions for nitrite and nitrate inducers and is only able to induce the recombinant protein under low oxygen pressure or anaerobic conditions.

## 2. Objectives

For this purpose and based on the native sequence of *E. coli* *nirB *promoter, we designed a synthetic *nirB *promoter and constructed a plasmid that it able to produce recombinant proteins under anaerobic induction and not by chemical inducers (nitrite or nitrate) and then the promoter activity was evaluated with regarding to the expression of a heterologous gene; heat-labile toxin B subunit (LTB gene), under various conditions.

## 3. Materials and Methods

### 3.1. Bacterial Strain, Enzymes and Reagents

*E. coli* DH5 was used in all cloning and expression procedures. Restriction enzymes, T4 DNA ligase, Taq DNA polymerase and dNTP were purchased from Fermentas (Lithuania). Ganglioside GMI type III, standard *E. coli* heat-labile toxin (LT) and anti-mouse horse-raddish peroxidase (HRP) conjugate antibodies were obtained from Sigma (USA). All cloning steps such as transformation, plasmid extraction, PCR amplification procedures and restriction digestion were performed on the basis of standard protocols (11).

### 3.2. PCR Amplification of LTB Gene

The LTB gene was generated by PCR on the pUCLTB plasmid, which was constructed as a template in our previous study (12). A 375-bp fragment was amplified using a forward primer containing NcoI site (underlined) 5′- GAATTCGGATGAAccATGgATAAAAG -3′ covering ATG translation initiation codon and a reverse primer 5′- CACAAGCTTCTAGTTTTCCATGATTG-3′ containing Hind III site (underlined).

### 3.3. Synthesis of nirB Promoter

The *nirB *synthetic promoter was designed based on the nitrite reductase gene sequence (X14202 gi: 42120) and constructed by two oligomers Bam*nirB*SD78; (5′ GATCCaggtaAATTTGATgTACATCAAatggtaccccttgctgaatcgt*TAAGGT*aggcggtaTaagGAGGaaaaaac) and Nco*NirB*SD78 (5′CATGGttttttcctccttataccgcctaccttaacgattcagcaaggggtaccatttgatgtacatcaaatttacctg). The two oligomers were mixed by molar ratio at 85 °C and cooled slowly to form a double-stranded fragment with cohesive ends of BamHI and NcoI sites at the 5′ and 3′ ends, respectively. On the Bam*nirB*SD78 oligo diagram, the FNR region has been shown by underlined capital letters, the ribosome-binding site (RBS) as bold letters and -10 region as italic capital letters.

### 3.4. Construction of pFSnirB78-23LTB

By two-step cloning, the synthetic *nirB *promoter (*nirB*: *Bam*HI- *Nco*I fragment) was firstly cloned in a pkk223 derivative plasmid and then the amplified LTB gene (LTB: *Nco*I-*Hind*III fragment) was cloned downstream of the promoter. This new construct was confirmed by restriction analysis and named pFS*nirB*78-23LTB.

### 3.5. Expression of Recombinant LTB Under Aerobic and Anaerobic Condition

The plasmid was transformed into competent *E. coli* and one colony of *E. coli*/ pFS*nirB*78-23LTB was cultured overnight in Luria-Bertani (LB) medium (yeast extract, peptone and NaCl) supplemented with ampicillin (100 ug/mL) at 37 C with vigorous shaking (220 rpm). For evaluation of promoter activity in aerobic conditions, 1/500 of overnight culture was inoculated into 5 mL of fresh medium containing ampicillin and incubated at 37 C with vigorous shaking (220 rpm) until OD_600 nm_ reached 0.4-0.6. Induction was carried out by adding sodium nitrite (2.5 mM) or sodium nitrate (20 mM), while a reaction without chemical induction was also performed; incubation was continued for 4 hours. The cells were then harvested and resuspended in 1 mL of PBS.

The colony forming unit (cfu) was determined by duplicate culture of 20 μL of each sample, which was serially diluted. The harvested cells were lysed by freezing and thawing three times with vigorous shaking and the crude lysate was analyzed for detection and measurement of the produced LTB by GM1-ELISA. The recombinant LTB concentration was calculated as ng/ 10^ 9^ cfu. Alternatively, for the reaction under anaerobic conditions, 1/30 of the overnight culture was inoculated into a 5 mL screw-cap conical tube completely filled with LB medium containing ampicillin. Sodium nitrite (2.5 mM) or sodium nitrate (20 mM) was added to each tube as an inducer for comparison with the control, which was kept under anaerobic conditions but did not contain the inducer. After 24 hours of incubation at 37 C, the cfu was determined and rLTB measurement was performed as mentioned above.

### 3.6. Ganglioside GM1 Enzyme Linked Immunosorbent Assay (GM1 ELISA)

The rLTB levels were determined and measured by the GM1 ELISA method as previously reported (13). Briefly, ELISA plates were coated with 5 μg ganglioside GM1 type III per well in 100 µL carbonate buffer, (pH 9.6) for 6 hours at room temperature. Next, the plates were blocked with PBS-0.1% bovine serum albumin (w/v) (PBS-BSA) at 37 C for 30 minutes. Diluted supernatants of the samples in PBS were added to each well in duplicate and after 1 hour of incubation at 37 C, the plates were washed 3 times with PBS-T (0.05% Tween20 in PBS). Anti-LTB/cholera toxin B subunit cross reactive monoclonal antibody D15-8 (kindly provided by the Pasteur Institute, Paris) and LT39 monoclonal antibody (kindly provided by Svennerholm, university of Goteborg, Sweden) were used as the first antibody and 100 µL of this diluted first antibody was added to each well and the plates were incubated at 37 C for 1 hour. The plates were then washed three times with PBS-T, and goat anti-mouse antibody conjugated with horseradish peroxidase (HRP) was added to each well. After 1 hour of incubation at room temperature followed by washing, chromogenic substrate, tetramethylbenzidine (TMB) and H_2_O_2_ were added to each well. The reaction was stopped by 2N H_2_SO_4_ after 15 minutes and absorbance was read at 450 nm. The amount of rLTB in each sample was determined by interpolation on standard curves generated using the reference LT. Finally, production rate of rLTB was calculated for 10^ 9^ cfu.

## 4. Results

**Figure 1.**
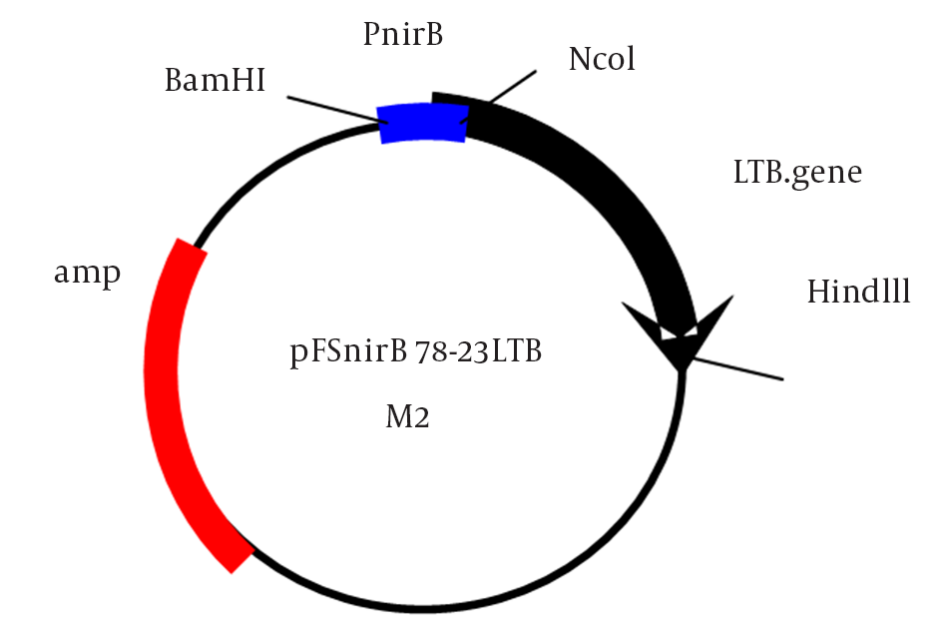
Schematic Construction of pFS*nirB*78-23LTB Plasmid

In this study, we constructed a new plasmid by two-step cloning as shown in Figure 1. At first, we cloned the 78 bp synthetic *nirB *promoter in a pKK223 derivative plasmid and then the LTB gene (375 bp) was cloned under the control of this promoter and the final plasmid was named pFS*nirB*78 -23LTB. The second codon of the native LTB gene was changed from AAT to GAT, which altered Asn to Asp in the second amino acid of the LTB signal peptide. The plasmid was transformed into *E. coli* DH5 in order to measure promotor activity following restriction analysis of the plasmid.

Activity of this promoter was determined by examining rLTB expression levels under both aerobic and anaerobic conditions and also the regulatory effect of chemical inducers nitrite and nitrate on the promoter was evaluated under both aerobic and anaerobic conditions. Our results showed that *E. coli*/ pFS*nirB*78-23 produces rLTB when anaerobiosis is solely the inducer. Under anaerobic conditions and also in the absence of any chemical inducer, the amount of rLTB was as high as 1560 ng per 109 cfu (Table 1). Chemical induction by both nitrite and nitrate had no more effect on increasing rLTB levels. Alternatively, under aerobic conditions the rLTB expression levels suppressed dramatically to as low as 11% of the anaerobic culture. However, supplementation of culture with nitrite and nitrate showed a greater decrease in rLTB expression levels.

**Table 1.** Comparision of rLTB Induction by *E. coli*/ pFS*nirB*78-23LTB Under Aerobic and Anaerobic Induction and Evaluation of Nitrite and Nitrate on Promoter Activity

	Expressed rLTB, ng/ 10^ 9^ cfu	
	Anaerobic	Aerobic
**Anaerobic induction**	1560	171
**Anaerobic induction + nitrite**	1600	79
**Anaerobic induction + nitrate**	1530	83

## 5. Discussion

Chemically inducible promoters such as isopropyl-beta-D-thiogalactopyranoside (IPTG) are extensively used for in vitro expression of recombinant proteins in bacteria. Due to the inducer’s stability, additional time and expensive steps to remove the inducers from the final product are required. Also, these promoters are not suitable for anaerobic conditions both in vitro and in vivo where there is not enough oxygen tension for cell growth. It seems that anaerobically inducible promoters such as the nirB promoter are an attractive alternative choice as they have successfully been used by numerous investigators. Various plasmids containing synthetic *nirB *promoters have been constructed and used for expression of foreign genes in *E. coli* (14, 15). These promoters have also been used for *in vivo* production of various antigens by *salmonella* live vaccine strains (16). 

**Figure 2.**
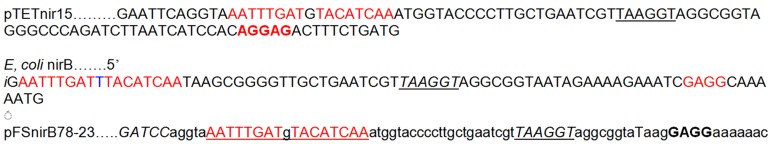
Comparison of Sequences of Intact Native *nirB *Promoter (GenBank gi:42120), pTETnir15 (14) and pFS*nirB*78-23 (this study) In pFS*nirB*78-23 plasmid the FNR region is visualized by underlined capital letters, ribosome-binding site (RBS) is shown by bold and -10 region by italic capital letters.

Intact native *nirB *promoter has also been used for expression of foreign genes in *Salmonella* live vaccine strains and has been shown to be an efficient system for immunization (17). With the aim of dissociation of chemical induciblity from anaerobiosis, we constructed an engineered synthetic *nirB *promoter, and evaluated the expression of LTB gene under anaerobic conditions, and determined the regulatory effects of nitrite and nitrate as chemical inducers on the activity of this promoter. We considered some critical regulatory regions on this engineered promoter. Firstly, we considered a hexamer sequence, TAAGGT at -10 position that is necessary for anaerobic activation. It has been shown that mutation in this region may result in loss of activity of the promoter (8). Secondly, a ribosome binding site (RBS) sequence was examined for control of translation. Thirdly, we evaluated the FNR global regulatory protein-binding site at -30 and -52, related to transcription start position. FNR protein encoded by the fnr gene regulates protein expression under anaerobic conditions via activation of transcription initiation of some anaerobic promoters such as *nirB *(7). We compared our *nirB *promoter with the intact native promoter and a synthetic promoter (pTETnir15) reported by Oxer et al. (14), as shown in Figure 2. There were some differences in the sequences that explain the probable variation between their activities.

Primarily, there were differences in the critical sequence for anaerobic activation; TAAGGT at -10 position upstream to the transcription start point and its distance to RBS. We selected this sequence so that it was exactly the same as the native promoter with slightly less distance to RBS. However, Oxer et al. preferred to choose the same sequence with more distance from RBS (14). The second critical region was the FNR protein-binding site between positions -52 and -30, upstream of the transcription start point. In P*nirB*78-23, this sequence is different from the native *nirB *promoter with respect to the central nucleotide but it is the same as that of pTETnir15 reported by Oxer et al. However, some investigations suggest more distance may not be important in expression rate. The third critical region is the RBS sequence and its distance to ATG translation initiation codon. 

We choose the TAAGGAGG sequence that it is exactly the same as the native promoter in four 3′ nucleotides but with more distance to the ATG codon. During the expression process, our data showed that the synthetic *nirB *promoter could be anaerobically induced and express rLTB in the *E. coli* host whereas it is extensively suppressed under aerobic conditions. Since growth of bacteria and induction of this promoter is different from other conventional expression systems, it might have adverse effects on the nature of the product. Assembling of LTB monomers in pentameric form (LTB5) is essential for binding LTB to its receptor (ganglioside GM1) and also for the immunogenicity and adjuvanticity of this molecule.

As shown by GM1-ELISA in this study, critical characteristics of the recombinant protein produced by this system such as pentamer formation, receptor recognition and antigenic epitopes are interestingly conserved. Reaction of LTB with LT39 mAb, a specific antibody for the pentamer but not the monomer form, resulted the correct structure formation of the produced LTB and conserved its receptor binding ability and immunogenic determinants. Our results are in agreement with those found by Newton and his colleagues that showed other foreign proteins could be efficiently expressed by the *nirB *promoter and well tolerated by *E. coli* (18). In *E. coli* FNR-dependent transcription is modulated by other regulatory systems (Nar/NarP), which appear to coordinate transcriptional responses with nitrate and nitrite. (9).

In native *nirB *promoter, the NarL-binding site is located at position -79 to -60 and contains an inverted repeat of two 10-base sequence elements. For removal of chemical induction from our construct, we didn’t consider this element in our synthetic promoter. As we expected our promoter did not respond to chemical inducers (nitrite and nitrate) of intact native *nirB *promoter (9). The findings of the present study showed that synthetic *nirB *can anaerobically produce foreign proteins, and removing the chemical inducibility of *nirB *promoter from its anaerobic activity is possible. In addition, anaerobiosis has no adverse effects on the produced protein. Accordingly, we think the *nirB *promoter is a suitable system for *in vitro* expression especially when high oxygen tension might have destructive effects on the recombinant protein. This system is potentially suitable for *in vivo* delivery of antigens or drugs by live bacterial strains such as *salmonella* live delivery vaccine strains.
